# Relationship between nutrition knowledge, education and other determinants of food intake and lifestyle habits among adolescents from urban and rural secondary schools in Tyrol, Western Austria

**DOI:** 10.1017/S1368980020000488

**Published:** 2020-12

**Authors:** Sabrina Egg, Maria Wakolbinger, Anna Reisser, Manuel Schätzer, Birgit Wild, Petra Rust

**Affiliations:** 1 Department of Dietetics, Health University of Applied Sciences, Innsbruck 6020, Austria; 2 Department of Nutritional Sciences, University of Vienna, Vienna 1090, Austria; 3 Department of Social and Preventive Medicine, Medical University of Vienna, Centre for Public Health, Vienna 1090, Austria; 4 Special Institute for Preventive Cardiology And Nutrition (SIPCAN), Elsbethen/Salzburg 5061, Austria; 5 Institute of Sports Medicine, Alpine Medicine and Health Tourism, Private University for Health Sciences, Medical Informatics and Technology GmbH (UMIT), Hall in Tirol 6060, Austria; 6 Institute for Vocational Education, Pedagogical University Tyrol, Innsbruck 6010, Austria

**Keywords:** Nutrition education, Nutrition knowledge, Dietary behaviour, Adolescents

## Abstract

**Objective:**

The aim of this study was to investigate the association of the number of hours of nutrition education and teachers’ qualifications with nutrition knowledge and dietary behaviour in students.

**Design:**

In this representative cross-sectional study, socio-demographic data, anthropometric measurements, socio-economic status (SES), physical fitness, nutrition knowledge and eating habits were assessed. Differences between groups were tested by *χ*^2^ and *t* tests. Multiple linear and logistic regression modelling was used to examine the relationship between demographic characteristics, lifestyle and dietary behaviours, nutrition knowledge, nutrition-trained teachers and number of nutrition lessons.

**Setting:**

Sixteen secondary schools in urban (*n* 6) and rural regions (*n* 10) of Tyrol, Western Austria.

**Participants:**

Students (*n* 513) aged 14·2 (sd 0·7) years.

**Results:**

Higher nutrition knowledge was significantly associated with attending rural school (*P* = 0·001), having no migration background (*P* < 0·001), (very) good physical activity behaviour (*P* = 0·040), non-trained teacher (*P* = 0·006) but higher number of hours of nutrition education (*P* = 0·013). Regression models showed that higher nutrition knowledge was independently associated with lower intake of meat and iced tea and higher intake of vegetables and plant-based oils. A higher amount of nutrition education (h/week) was significantly associated with higher intake of dark (wholegrain) bread, lower intake of meat and of energy drinks sweetened with sweeteners.

**Conclusions:**

Our results suggest that more hours in nutrition education result in higher nutrition knowledge and greater nutrition literacy, which may lead to health-promoting dietary habits. School-based nutrition education can be seen as preventive measure to increase nutritional competences in adolescents independent of their SES.

Due to the rise of overweight and obesity, dietary intake, especially in children and adolescents, has been of great concern in previous years. Worldwide, the prevalence of overweight and obesity in children and adolescents has risen from 4 to 18 % between 1975 and 2016. Consequently, the mean BMI has increased during these years^(1,2)^. According to the German Health Interview and Examination Survey for Children and Adolescents, the number of overweight and obese children and adolescents aged 3–17 years has increased up to 50 % during the past 30 years^(3)^. Also, in Austria, overweight and obesity have long been on the rise. A representative survey from 2017 has shown that 26 % of 8- to 9-year-old boys and 25 % of the girls are overweight or obese^(4)^ compared with data from 2008 where the prevalence of overweight/obesity was 21 % in boys and 17 % in girls^(5)^. Due to associated comorbidities and the impact of obesity on the healthcare system, the WHO speaks of one of the greatest public health challenges of the 21st century^(2)^. On the other hand, the Organisation for Economic Co-operation and Development (OECD) report 2018 found reverse tendencies regarding the obesity rate in 7- to 8-year-old children between the survey periods in 2007/2008 and 2015/2017^(6)^. Still, on average, nearly one in eight children in EU countries is obese, and therefore, obesity is remaining one of the main health problems in many countries.

Numerous studies have found that the majority of children do not meet the recommendations of food-based dietary guidelines and energy and nutrient intake, mostly showing a lower fruit and vegetable intake but higher meat and sugar consumption, resulting in high intakes of SFA and salt, and low intakes of PUFA, vitamin D, folate and iodine^(7,8)^, especially in Western countries^(9–13)^. Dietary habits are established early in life and maintain into adulthood^(14–16)^. This represents an issue, especially with their potential lifelong effects on the development of chronic diseases such as obesity, hypertension, CHD, diabetes mellitus, certain types of cancer and thus, premature mortality and physical morbidity^(17–22)^. Freedman *et al*.^(23)^ found that nearly 70 % of obese adolescents have at least one risk factor for CVD. Therefore, childhood and adolescence are a period where good nutritional quality is important for the establishment of health-promoting eating habits that can prevent the development of nutrition-associated health disorders later in life.

Reasons for the rise of obesity in children and adolescents are diverse and include poor food choices, physical inactivity, sedentary lifestyle, peer groups, family structure and socio-economic status (SES) of the parents^(24–29)^. Furthermore, some literature suggests that nutrition knowledge in children and young adolescents may have an impact on dietary habits^(30)^. In Europe^(24)^, including Austria^(31)^, poor nutrition knowledge in adolescents was found and may be associated with overweight and obesity. On the other hand, studies have demonstrated that sufficient knowledge is not necessarily associated with health-promoting dietary behaviour due to several other psychological and environmental factors that might play a role^(32,33)^.

Therefore, with the rising number of overweight and obese children, the WHO included nutrition education in their European Core Curriculum^(34)^. The aim was not only to communicate nutritional knowledge but also to develop skills and behaviours related to food preparation, preservation and storage, social and cultural aspects of food and nutrition, as well as improving self-esteem for the development of a positive body image, and thus, improve nutrition literacy. All these aspects are conducive to make healthy food choices and develop lifelong health-promoting eating patterns^(34)^. According to several studies, dietary and physical interventions in combination with structural interventions can result in healthier dietary behaviour among schoolchildren^(35–39)^. However, few studies have examined the direct relationship between school nutrition education as a fixed part of the curriculum and dietary behaviour. Those studies suggest that holistic nutrition education, which takes into account the factors mentioned above, can lead to healthier dietary behaviour among schoolchildren^(40,41)^.

Nutrition education in Germany and Austria has found little room in the curricula and timetables so far. It is often included as part of other subjects or implemented in the context of temporary project days or weeks. Although being recommended on the European level, implementation is slow, if not even on hold, in terms of educational policy^(42)^. In Austria with the exception of secondary schools with certain specialisations, only the so-called ‘New Middle Schools’ (NMS) include ‘Nutrition and Household’ as an independent subject within the curriculum, with each school autonomously deciding how many hours per week should be spent, with one lesson (50 min) per week being the minimum and four lessons the maximum^(43)^.

There is yet no literature regarding the association of teacher training and the number of hours of nutrition education and changes in dietary behaviour in children. However, a review investigating the association between teacher training and behaviour change regarding physical activity found that teachers are capable of making substantial improvements in student outcomes in physical activity. However, the findings of this review suggest that the impact of teacher training on school-based interventions is not only under-reported but also under-studied^(44)^.

Despite a well-elaborated curriculum^(45)^, budget limitations, time constraints and the lack of trained teachers or rather the fair distribution of trained teachers to all schools represent current challenges regarding nutrition education. Therefore, the aim of this study was to investigate the association of the number of hours of nutrition education offered in the curriculum and the qualification of the teachers who teach the subject ‘Nutrition and Household’ with nutrition knowledge and dietary behaviour in 14- and 15-year-old students in Tyrol, Western Austria.

## Methods

### Participants and procedure

**Fig. 1:**
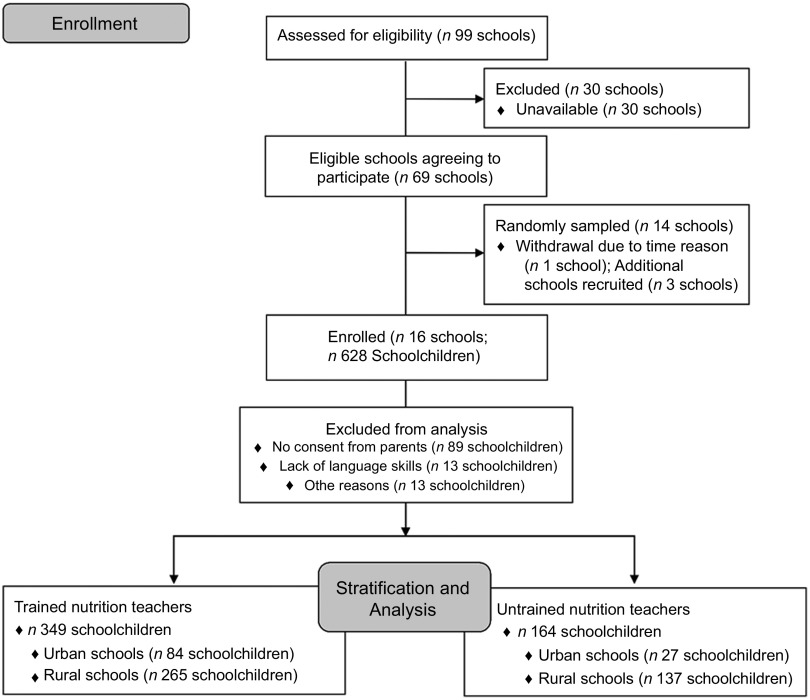
Flow chart of participant recruitment

This cross-sectional study was carried out in the eighth grade of sixteen randomly selected NMS in the federal state Tyrol, Western Austria. In order to obtain representative results for Tyrol, a minimum net sample of 500 subjects was required with a significance level of ±5 % (*P* < 0·05), within a 95 % CI for a population of 5231 students in the school year 2015/2016. Based on the information provided by the School Board, which included ninety-nine NMS in Tyrol, as well as information about the qualification of the teachers, the schools were stratified by teachers’ qualification, of which seven schools were selected with untrained nutrition teachers, and then stratified a second time by the location of the schools (seven urban and seven rural schools). Therefore, fourteen NMS were randomly sampled with the aim to reach approximately 500 schoolchildren from the eighth grade. As participation in the survey was rejected by one NMS because of time reasons, another three NMS were selected and contacted, of which all accepted to participate (Fig. 1). Appointments were organised in agreement with the schools’ principals. Since the survey was anonymous and no identifying data of the students were collected, consent of the parents was waived. The legal guardians of the students, however, had the opportunity to refuse their child’s participation in the survey by signing a declaration of non-consent.

The data collection took place in June 2016. The final random sample included sixteen NMS in Tyrol with a total of 628 students. Response rate was 81·7 % resulting in 115 (18·3 %) students who did not participate in the data collection with following reasons: eighty-nine students (77·4 %) did not participate because they or their parents did not want personal data collection, thirteen students (11·3 %) with migration background declined to participate because of lacking language skills and religious reasons and another thirteen students (11·3 %) refused to participate without naming any reasons.

### Data collection and material

Data were collected during a pre-organised visit to each school. The students completed the questionnaire in class and in the presence of two trained dietitians and nutritional scientists who guided them through the questionnaire and explained every question in order to avoid misunderstandings and mistakes. The questionnaire was divided into six sections.

The first section comprised four questions about the subject ‘Nutrition and Household’. Two questions referred to the number of lessons per week and school year as well as the qualification of the teachers for the subject and were answered by the schools’ principals beforehand. Additionally, we assessed the number of sports lessons. The students completed the two remaining questions about their personal meaning of practical classes and possible personal benefits from the subject.

The second section covered socio-demographic data and included five questions regarding age, gender and migration background.

The third section included four questions regarding socio-economic data and was based on the validated Family Affluence Scale^(46)^. The scale comprises four items including the number of cars and computers within the household, own bedroom and the number of family vacations within the previous year. Family Affluence Scale indicates the SES of the students on a scale from 0 (very low SES) to 8 (very high SES). Following the example of Rey-López *et al*.^(47)^, we recoded the scores as low SES (0–2 points), medium SES (3–5 points) and high SES (6–8 points).

The fourth section aimed to measure the level of physical fitness with the validated International Fitness Scale^(48)^ which included five questions regarding general physical fitness, cardiorespiratory fitness, muscular strength, speed/agility and flexibility. Scores were built and classified as very good (21–25 points), good (16–20 points), sufficient (11–15 points), bad (6–10 points) and very bad (1–5 points)^(48)^.

Furthermore, the children self-reported the usual number of hours they spend per day in front of the TV, computer and mobile phone.

The fifth section comprised questions on nutrition knowledge based on the current Austrian guidelines^(49)^ and on the curriculum for the subject ‘Nutrition and Household’, which defines the minimum goals for this subject. On the one hand, recommended consumption rates of individual foods or food groups were inquired; on the other hand, questions about correct and incorrect food and drink statements were asked. We calculated the percentage of correct answers for the age-speciﬁc nutrition knowledge questionnaire, based on previous studies^(30,50)^.

The sixth and last section contained a semi-quantitative self-administered FFQ, which is based on the FFQ used for the Austrian Study on Nutritional Status^(51)^ to assess the children’s dietary behaviour. The FFQ comprised twenty-four food items including vegetables, salad, pulses, nuts and seeds, fruits, potatoes, cereals and cereal products, bread (white bread and wholegrain), dairy and dairy products (yogurt, cheese), meat and meat products, fish, eggs, animal fats (like butter), plant oils, fast food, salty snacks and sweets. Drinks were assessed including seventeen items: water, fruit juice, diluted fruit juice, smoothies, fruit nectar, beverages (separated in ice tea, sugar sweetened or light), energy drinks, tea and coffee (with and without sugar). One of the main inaccuracies in dietary intake estimation by using an FFQ is portion size, which is the reason why pictures were included to visualise portion sizes (hand or cup) for each item. The seven frequency response options, ranging from ‘never’ to ‘two times or more per day’, were transformed into daily frequency of consumption.

### Anthropometry

Anthropometric measurements, such as body height, body weight and waist circumference, were determined for each student by two trained dietitians and nutritional scientists. Body height was assessed in centimetres with shoes off by means of a measuring tape with measuring accuracy of 0·1 cm. Body weight was measured in kilograms using a mobile calibrated digital scale (Marsden MS-4203^®^) with an accuracy of 0·1 kg with light clothing and shoes off. BMI was calculated by dividing the weight by square of height (kg/m^2^). To assess the prevalence of overweight and obesity, the gender- and age-specific limits of the WHO^(52,53)^ were used. Waist circumference was measured in centimetres by means of an ergonomic, extendable measuring tape (*Kleiber*) with a measuring accuracy of 0·1 cm, which was applied at the midpoint of the lower rib border and the iliac crest, after expiration^(54)^.

### Statistical analyses

Sample characteristics are presented as frequencies or mean and sd as appropriate. Differences between groups were tested by the *χ*^2^ test for categorical variables, while for continuous variables, parametric (Student’s *t*) tests were used.

The internal consistency of the percentage of correct answers for the age-speciﬁc nutrition knowledge questionnaire was evaluated using Cronbach’s *α*^(55)^. The closer *α* is to one, the higher is the reliability estimate of the instrument. Nutrition knowledge was first described in four levels (quartiles) of the adolescents’ nutrition knowledge groups (low, medium–low, medium–high and high) which were then categorised into two groups: low–medium and medium–high.

Multiple linear and logistic regression modelling was used to examine the relationship between selected demographic characteristics, lifestyle and dietary behaviours and nutrition knowledge, nutrition-trained teachers, nutrition and sports lessons, adjusted for covariates such as urban/rural region, age, gender, migration background and SES. Statistical analyses were performed with IBM^®^ SPSS^®^ Statistics for Windows, version 25 software (IBM Corp.). *P* values <0·05 were considered statistically significant, and all tests were two-sided.

## Results

### Characteristics of the study participants

A total of 513 schoolchildren at sixteen secondary schools were included in this cross-sectional study. This is a representative sample consisting of six urban and ten rural schools. Demographic characteristics and lifestyle behaviours are presented in Table 1. The mean age of participants was 14·2 (sd 0·7) years (range 13–18 years). Gender was approximately equal in representation, with boys accounting for slightly more of the participants (51 %). Boys were signiﬁcantly more likely to be overweight or obese (*P* = 0·042) with higher waist circumference (*P* < 0·001) but a higher percentage of self-reported (very) good physical activity behaviour (*P* < 0·001) and were older (*P* = 0·050).

**Table 1:** Sample characteristics: adolescents (*n* 513) in secondary schools in Tyrol, Western Austria

Characteristics	Boys (*n* 263)			Girls (*n* 250)			*P* *	Total (*n* 513)		
	*n*		%	*n*		%		*n*		%
Age (years)										
Mean		14·2			14·1		**0·050**		14·2	
sd		0·8			0·6				0·7	
Height (cm)										
Mean		171·2			162·6		**<0·001**		167·0	
sd		8·0			6·5				8·4	
Body weight (kg)										
Mean		64·5			57·7		**<0·001**		61·2	
sd		14·8			11·1				13·5	
Waist circumference (cm)										
Mean		74·9			70·6		**<0·001**		72·8	
sd		10·7			8·7				10·0	
BMI (kg/m^2^)										
Mean		21·9			21·8		0·778		21·8	
sd		4·3			3·8				4·0	
BMI classification										
Underweight	2		0·8	1		0·4	**0·042**	3		0·6
Normal weight	148		58·3	167		69·6		315		63·8
Overweight	68		26·8	53		22·1		121		24·5
Obese	36		14·2	19		7·9		55		11·1
Migration background										
No	149		56·7	141		56·4	0·954	290		56·5
Yes	114		43·3	109		43·6		223		43·5
Socio-economic status										
Low	5		1·9	6		2·4	0·506	11		2·1
Medium	78		29·7	63		25·2		141		27·5
High	180		68·4	181		72·4		361		70·4
Physical activity behaviour										
(Very) poor	4		1·5	6		2·4	**<0·001**	10		2·0
Adequate	32		12·2	40		16·1		72		14·1
Good	107		40·8	145		58·2		252		49·3
Very good	119		45·4	58		23·3		177		34·6
Media consumption (h/d)										
Mean		9·7			10·4		0·088		10·1	
sd		7·2			7·0				7·1	
Nutrition knowledge (% of correct answers)	49·2		14·1	49·6		13·4	0·719	49·4		13·7

* Independent samples *t* tests or *χ*^2^ test, as appropriate. Signiﬁcant ﬁndings are bold.

### Nutrition knowledge

The mean percentage of correct answers of the nutrition knowledge questionnaire was 49·4 % (13·7), 45·5 % (14·1) among adolescents in urban schools, 50·5 % (13·5) among those in rural schools, 45·9 % (14·5) among those with migration background and 52·1 % (12·5) among those without migration background. Internal consistency measured by the Cronbach’s *α* for the nutrition knowledge questionnaire was 0·490 (in total), 0·505 (urban), 0·473 (rural), 0·537 (migration background) and 0·395 (no migration background). Findings regarding the association between demographic characteristics, lifestyle and dietary behaviours and nutrition knowledge are reported in Table 2. Means and sd of nutrition knowledge (in percentage of correct answers) are shown to highlight demographic and lifestyle differences. Higher nutrition knowledge scores were significantly associated with being in a rural region or school, respectively (*P* = 0·001), having no migration background (*P* < 0·001), having (very) good physical activity behaviour (*P* = 0·040), having a non-trained teacher (*P* = 0·006) but having a higher amount of nutrition education (h/week; *P* = 0·013).

**Table 2:** Distribution of nutrition knowledge in percentage of correct answers according to demographic characteristics and lifestyle behaviours of the study: adolescents (*n* 513) aged 13–14 years in urban and rural schools of Tyrol, Western Austria

Variable	Nutrition knowledge (% of correct answer)		*P* *
	Mean	sd	
Gender			
Male	49·2	14·1	0·719
Female	49·6	13·4	
Region			
Urban	45·5	14·1	**0·001**
Rural	50·5	13·5	
BMI classification (%)			
Under and normal weight	49·4	13·6	0·598
Overweight	49·4	13·4	
Obese	51·3	13·3	
Migration background			
No	52·1	12·5	**<0·001**
Yes	45·9	14·5	
Socio-economic status			
Low	46·8	8·4	0·084
Medium	47·2	15·0	
High	50·3	13·3	
Physical activity behaviour			
(Very) poor	42·0	8·6	**0·029**
Adequate	46·5	14·5	
Good	49·9	13·3	
Very good	50·3	14·2	
Having trained teacher			
No	51·7	12·3	**0·006**
Yes	48·3	14·3	
Nutrition education			
<2·5 h/week	48·4	14·2	**0·040**
≥2·5 h/week	51·0	12·9	

* Independent samples *t* tests or *χ*^2^ test, as appropriate. Signiﬁcant ﬁndings are bold.

The relationship between the adolescent’s nutrition knowledge and their demographic characteristics, lifestyle and dietary behaviours is shown in Table 3. A higher knowledge level was significantly associated with higher body weight, higher intake of vegetables and plant-based oils (model 2, adjusted for urban/rural region, age, gender, migration background and SES). Media consumption, the intake of meat, salty snacks, fat-free milk, iced tea and energy drinks (sweetened with sweeteners) were negatively associated with nutrition knowledge (model 2, adjusted for urban/rural region, age, gender, migration background and SES).

**Table 3:** Relationship between selected demographic characteristics, lifestyle and dietary behaviours (frequency/d) and nutrition knowledge in percentage of correct answers, nutrition and sports lessons (h/week) by linear regression analysis: adolescents (*n* 513) aged 13–14 years in urban and rural schools of Tyrol, Western Austria

Dependent variables	Independent variables	Model 1*		Model 2†	
		*β*	*P*	*β*	*P*
Media consumption (h/week)	Nutrition knowledge (%)	–0·200	**<0·001**	–0·093	**0·039**
	Nutrition lessons (h/week)	–0·085	**0·028**	–0·077	0·058
	Sports lessons (h/week)	0·180	**<0·001**	0·119	**0·009**
Physical activity behaviour (score)	Nutrition knowledge (%)	0·068	0·124	0·080	0·078
	Nutrition lessons (h/week)	0·032	0·406	–	**–**
	Sports lessons (h/week)	0·050	0·252	–	**–**
Weight (kg)	Nutrition knowledge (%)	0·118	**0·010**	0·128	**0·006**
	Nutrition lessons (h/week)	0·022	0·588	–	–
	Sports lessons (h/week)	–0·033	**0·470**	0·235	**0·014**
Waist circumference (cm)	Nutrition knowledge (%)	0·079	0·076	–	–
	Nutrition lessons (h/week)	0·028	0·475	–	–
	Sports lessons (h/week)	–0·096	**0·033**	–0·302	**0·001**
Frequency of consumption of… (per day)					
Dark (wholegrain) bread	Nutrition knowledge (%)	0·083	0·052	–	–
	Nutrition lessons (h/week)	0·110	**0·003**	0·100	**0·009**
	Sports lessons (h/week)	–0·118	**0·005**	–0·101	**0·018**
Meat	Nutrition knowledge (%)	–0·152	**0·001**	–0·082	0·078
	Nutrition lessons (h/week)	–0·089	**0·020**	–0·089	**0·027**
	Sports lessons (h/week)	0·060	0·165	–	–
Vegetables	Nutrition knowledge (%)	0·172	**<0·001**	0·103	**0·021**
	Nutrition lessons (h/week)	0·042	0·261	–	–
	Sports lessons (h/week)	–0·085	**0·047**	–	–
Fast food	Nutrition knowledge (%)	–0·177	**<0·001**	–	–
	Nutrition lessons (h/week)	–0·040	0·291	–	–
	Sports lessons (h/week)	0·125	**0·004**	–	–
Salty snacks	Nutrition knowledge (%)	–0·193	**<0·001**	–0·115	**0·026**
	Nutrition lessons (h/week)	–0·053	0·159	–	–
	Sports lessons (h/week)	0·092	**0·032**	–	–
Milk (fat-free)	Nutrition knowledge (%)	–0·090	**0·036**	–0·070	0·101
	Nutrition lessons (h/week)	–0·071	0·057	–	–
	Sports lessons (h/week)	0·094	**0·027**	0·078	0·073
Whey	Nutrition knowledge (%)	–0·074	0·085	–	–
	Nutrition lessons (h/week)	–0·041	0·274	–	–
	Sports lessons (h/week)	0·125	**0·003**	0·131	**0·003**
Oil	Nutrition knowledge (%)	0·119	**0·005**	0·117	**0·009**
	Nutrition lessons (h/week)	–0·006	0·878	–	–
	Sports lessons (h/week)	0·008	0·856	–	–
Iced tea	Nutrition knowledge (%)	–0·226	**<0·001**	–0·134	**0·007**
	Nutrition lessons (h/week)	0·019	0·631	0·072	0·087
	Sports lessons (h/week)	0·033	0·449	–0·092	0·090
Diet drinks	Nutrition knowledge (%)	–0·137	**0·002**	–	–
	Nutrition lessons (h/week)	–0·058	0·126	–	–
	Sports lessons (h/week)	0·108	**0·012**	–	–
Soft drinks	Nutrition knowledge (%)	–0·158	**<0·001**	–	–
	Nutrition lessons (h/week)	–0·025	0·516		
	Sports lessons (h/week)	0·087	**0·044**	0·080	0·118
Energy drinks with sugar	Nutrition knowledge (%)	–0·121	**0·007**	–	–
	Nutrition lessons (h/week)	–0·079	**0·041**	–	–
	Sports lessons (h/week)	0·116	**0·008**	–	–
Energy drinks with sweeteners	Nutrition knowledge (%)	–0·134	**0·002**	–0·070	0·105
	Nutrition lessons (h/week)	–0·067	0·077	–0·082	**0·035**
	Sports lessons (h/week)	0·100	**0·019**	0·102	**0·021**

SES, socio-economic status. Signiﬁcant ﬁndings are bold.

* Linear regression model was used to examine the relationship between demographic characteristics, lifestyle and dietary behaviours (frequency/d) and nutrition knowledge in percentage of correct answers, nutrition and sports lessons (h/week). Model 1: Univariate linear regression adjusted for urban/rural region, age, gender, migration background and SES.

† Model 2: Multivariate linear regression with backward selection adjusted for urban/rural region, age, gender, migration background and SES.

Multiple logistic regression modelling was used to assess the association between nutrition knowledge and demographic characteristics, lifestyle and dietary behaviours. Regression analysis revealed that nutrition knowledge was independently associated with lower intake of meat and iced tea, and a higher intake of vegetables and plant-based oils (Table 4, adjusted for urban/rural region, age, gender, migration background and SES).

**Table 4:** Relationship between selected demographic characteristics, lifestyle and dietary behaviours (frequency/d) and nutrition knowledge (cut-off ≥55 %, medium/high), nutrition-trained teacher, and nutrition lessons (≥2·5 h/week) by logistic regression analysis: adolescents (*n* 513) aged 13–14 years in urban and rural schools of Tyrol, Western Austria, 2016

Dependent variables	Independent variables	Model 1*				Model 2†			
		OR	95 % CI		*P*	OR	95 % CI		*P*
Media consumption (h/week)	Medium/high nutrition knowledge	0·945	0·916	0·974	**<0·001**	0·975	0·941	1·010	0·162
	Nutrition-trained teacher	1·038	1·005	1·072	**0·022**	1·031	0·997	1·066	0·073
	Nutrition lessons ≥2·5 h/week.	0·977	0·948	1·008	0·144	–	–	–	–
Physical activity behaviour (score)	Medium/high nutrition knowledge	1·422	1·092	1·851	**0·009**	1·315	0·958	1·804	0·090
	Nutrition-trained teacher	0·901	0·686	1·183	0·453	–	–	–	–
	Nutrition lessons ≥2·5 h/week.	1·025	0·777	1·353	0·862	–	–	–	–
Weight (kg)	Medium/high nutrition knowledge	1·007	0·992	1·021	0·360	1·029	0·995	1·064	0·091
	Nutrition-trained teacher	1·009	0·993	1·024	0·273	–	–	–	–
	Nutrition lessons ≥2·5 h/week.	1·007	0·991	1·023	0·391	–	–	–	–
Waist circumference (cm)	Medium/high nutrition knowledge	0·999	0·981	1·019	0·956	0·965	0·922	1·009	0·117
	Nutrition-trained teacher	1·006	0·986	1·027	0·563	–	–	–	–
	Nutrition lessons ≥2·5 h/week.	1·011	0·990	1·032	0·304	–	–	–	–
Frequency of consumption of … (per day)									
Dark (wholegrain) bread	Medium/high nutrition knowledge	1·426	1·040	1·955	**0·027**	–	–	–	–
	Nutrition-trained teacher	1·085	0·777	1·515	0·634	–	–	–	–
	Nutrition lessons ≥2·5 h/week.	1·755	1·240	2·484	**0·002**	1·664	1·162	2·384	**0·005**
Meat	Medium/high nutrition knowledge	0·466	0·306	0·711	**<0·001**	0·467	0·283	0·773	**0·003**
	Nutrition-trained teacher	0·958	0·642	1·429	0·834	–	–	–	–
	Nutrition lessons ≥2·5 h/week.	0·619	0·410	0·934	0·022	0·689	0·442	1·074	0·100
Vegetables	Medium/high nutrition knowledge	1·908	1·360	2·678	**<0·001**	1·522	1·005	2·303	**0·047**
	Nutrition-trained teacher	0·949	0·673	1·337	0·764	–	–	–	–
	Nutrition lessons ≥2·5 h/week.	1·178	0·817	1·698	0·379	–	–	–	–
Legumes	Medium/high nutrition knowledge	2·289	1·194	4·390	**0·013**	1·751	0·790	3·880	0·168
	Nutrition-trained teacher	0·509	0·272	0·953	**0·035**	0·559	0·294	1·063	0·076
	Nutrition lessons ≥2·5 h/week.	1·016	0·500	2·064	0·966	–	–	–	–
Fast food	Medium/high nutrition knowledge	0·308	0·162	0·587	**<0·001**	–	–	–	–
	Nutrition-trained teacher	1·609	0·899	2·877	0·109	0·594	0·294	2·950	0·215
	Nutrition lessons ≥2·5 h/week.	0·685	0·399	1·177	0·171	–	–	–	–
Salty snacks	Medium/high nutrition knowledge	0·377	0·228	0·623	**<0·001**	0·553	0·292	1·047	0·069
	Nutrition-trained teacher	0·936	0·603	1·454	0·769	–	–	–	–
	Nutrition lessons ≥2·5 h/week.	0·687	0·422	1·120	0·132	–	–	–	–
Milk (fat-free)	Medium/high nutrition knowledge	0·676	0·355	1·288	0·234	0·571	0·302	1·080	0·085
	Nutrition-trained teacher	0·958	0·500	1·835	0·897	–	–	–	–
	Nutrition lessons ≥2·5 h/week.	0·382	0·167	0·872	**0·022**	0·402	0·165	0·979	**0·045**
Whey	Medium/high nutrition knowledge	0·878	0·467	1·649	0·685	–	–	–	–
	Nutrition-trained teacher	0·909	0·475	1·739	0·773	–	–	–	–
	Nutrition lessons ≥2·5 h/week.	0·510	0·233	1·115	0·092	0·538	0·225	1·287	0·164
Oil	Medium/high nutrition knowledge	1·603	1·111	2·312	0·012	1·789	1·129	2·833	**0·013**
	Nutrition-trained teacher	0·951	0·651	1·389	0·796	–	–	–	–
	Nutrition lessons ≥2·5 h/week.	1·086	0·731	1·613	0·683	–	–	–	–
Iced tea	Medium/high nutrition knowledge	0·325	0·202	0·523	**<0·001**	0·396	0·225	0·699	**0·001**
	Nutrition-trained teacher	1·125	0·747	1·693	0·574	–	–	–	–
	Nutrition lessons ≥2·5 h/week.	1·093	0·712	1·678	0·685	1·571	0·945	2·612	0·081
Diet drinks	Medium/high nutrition knowledge	0·482	0·224	1·037	0·062	–	–	–	–
	Nutrition-trained teacher	0·762	0·399	1·456	0·410	0·594	0·299	1·181	0·137
	Nutrition lessons ≥2·5 h/week.	0·523	0·241	1·135	0·101	–	–	–	–
Soft drinks	Medium/high nutrition knowledge	0·391	0·233	0·658	**<0·001**	–	–	–	–
	Nutrition-trained teacher	0·937	0·588	1·493	0·784	–	–	–	–
	Nutrition lessons ≥2·5 h/week.	0·788	0·471	1·317	0·363	–	–	–	–
Energy drinks with sugar	Medium/high nutrition knowledge	0·688	0·440	1·077	0·102	1·683	0·947	2·991	0·076
	Nutrition-trained teacher	1·133	0·712	1·801	0·599	–	–	–	–
	Nutrition lessons ≥2·5 h/week.	0·551	0·328	0·925	0·024	0·590	0·319	1·093	0·093
Energy drinks with sweeteners	Medium/high nutrition knowledge	0·734	0·382	1·407	0·351	–	–	–	–
	Nutrition-trained teacher	0·942	0·500	1·774	0·853	–	–	–	–
	Nutrition lessons ≥2·5 h/week.	0·516	0·247	1·078	0·079	–	–	–	–

SES, socio-economic status. Signiﬁcant ﬁndings are bold.

* Logistic regression model was used to examine the relationship between demographic characteristics, lifestyle and dietary behaviours (frequency/d) and nutrition knowledge (medium–high *v*. low–medium), nutrition-trained teacher (yes *v*. no), and nutrition lessons (≥2·5 *v*. <2·5 h/week). Model 1: Univariate logistic regression adjusted for urban/rural region, age, gender, migration background and SES.

† Model 2: Multivariate logistic regression with backward selection adjusted for urban/rural region, age, gender, migration background and SES.

### Nutrition education

The mean number of hours in nutrition education was 2·3 (0·7) h/week, 1·6 (0·4) h among adolescents in urban schools and 2·6 (0·7) h among those in rural schools (*P* < 0·001). A higher amount of nutrition education was significantly associated with higher intake of dark (wholegrain) bread, a lower intake of meat and of energy drinks sweetened with sweeteners (Table 3; model 2, adjusted for urban/rural region, age, gender, migration background and SES).

Logistic regression analysis revealed that the odds of consuming dark (wholegrain) bread (OR 1·7) increased while the odds of consuming fat-free milk (OR 0·4) decreased as the number of nutrition lessons increased (≥2·5 h/week; Table 4; model 2, adjusted for urban/rural region, age, gender, migration background and SES).

In terms of the nutrition training of the adolescent’s teachers, we found no significant associations with items regarding dietary behaviour by considering the cohort as a whole. Given the regional differences found, we divided the cohort into two subgroups: (i) adolescents in urban schools and (ii) those in rural schools. Applying such a division in the aforementioned logistic regression analysis revealed following results: In those adolescents with a trained teacher and living in an urban region, the odds of consuming vegetables (OR 3·78, 95 % CI 1·06, 13·46; *P* = 0·040) increased, while the odds of a higher waist circumference decreased (OR 0·87, 95 % CI 0·76, 0·99; *P* = 0·043, adjusted for age, gender, migration background and SES). We found no significant impact on dietary behaviour in adolescents living in a rural region.

## Discussion

In the current study, with a cross-sectional design, we aimed to assess associations between nutritional knowledge, food intake and the amount of lessons in nutrition education as well as the presence of trained teachers for the subject ‘Nutrition and Household’ among children and young adolescents in secondary schools (NMS) in Tyrol with the consideration of demographic and socio-economic data. Our data show associations of higher nutrition knowledge with being in a rural school, having no migration background, having (very) good physical activity behaviour (self-reported), having a non-trained teacher but having a higher amount of nutrition education hours per week. Our results also show a tendency of a higher SES being associated with higher nutrition knowledge. To date and to our knowledge, the current study is the first one assessing these associations in a representative sample of adolescents living in rural and urban regions of Tyrol, Western Austria.

Studies found that children with a migration background are at higher risk for overweight^(56,57)^, one of them, being a German study, demonstrated a higher prevalence of overweight and obesity among children with a migration background^(3)^. According to the authors, this might be due to the higher consumption of foods rich in sugar and fat^(57)^. However, in an experimental study, Weber *et al*.^(58)^ could improve the children’s short-term nutrition knowledge and skills by providing practical nutrition lessons in primary schools with a high proportion of children with a migration background. Another similar study by Kaufmann-Shriqui *et al*.^(59)^ in school children with low SES (which often is associated with migration background and consuming poor diets^(60)^) reached promising results with a school-based intervention not only on nutrition knowledge but also on dietary habits. The intervention included nutrition classes for children, mothers and teachers within a duration of 6 months. Eating habits and nutrition knowledge, as well as the quality of their packed lunches, improved significantly. This intervention highlights the impact of involving mothers and teachers in health promotion activities and that nutritional education programmes as part of the school curriculum can contribute to maintaining a healthy body weight of schoolchildren.

When adjusting a healthy lifestyle, good nutrition and regular physical activity are essential. Several studies exist where both aspects are considered as part of an intervention programme^(56,61)^. Despite the knowledge about adequate nutrition and physical activity, the challenge is its application. Knowledge about nutrition and physical activity often improves but not the behaviour^(62)^. Still, our study somewhat shows an association between higher nutrition knowledge and higher physical activity, although these results need to be carefully looked at since variables about physical activity rely on self-reported responses. However, these heterogenic results imply that numerous factors play a crucial role in the development of a healthy lifestyle, one of them is parental involvement in school interventions since they act as role models and schoolchildren are mostly dependent on the food and activity choices their parents make at home^(30,63,64)^.

In addition, we found associations between higher nutrition knowledge and higher body weight but healthier food choices. However, the associations with BMI or waist circumference were not significant. Media consumption, the intake of meat, salty snacks, fat-free milk, iced tea and energy drinks (sweetened with sweeteners) were negatively associated with nutrition knowledge. In addition, we observed that a higher knowledge level was associated with higher vegetable and oil consumption. Results in comparable studies show a positive impact of knowledge on food consumption and nutritional behaviour in children and adolescents: Intake of foods considered to be healthy, such as vegetables, fruits and starchy foods, was often higher when nutrition knowledge was higher, while consumption of unhealthy foods such as sweets, snacks, fried foods and sugary drinks was negatively associated with nutrition knowledge^(7,30)^. Similar to our study, studies have also shown a significant difference between boys and girls, demonstrating boys having lower nutrition knowledge and lower consumption of vegetables and fruits but a higher consumption of meats, fats and soft drinks compared with girls^(7,65,66)^. On the other hand, there are also several studies with no significant associations between nutrition knowledge and dietary behaviour, which confirms again that dietary behaviour and lifestyle are a multifactorial result of various influences of psychological, behavioural, genetic, environmental, socio-economic and socio-demographic origin^(32,33)^.

Although hypothesised contrarywise, we found that children having a trained teacher demonstrated lower nutrition knowledge compared with those with non-trained teachers. However, a higher amount of nutrition lessons per week was associated with higher nutrition knowledge. Consequently, the number of nutrition lessons per week in the curriculum seems to have a greater impact on nutrition knowledge than the qualification of teachers in nutrition. Moreover, our findings demonstrate a significant difference between urban and rural schools, with more hours of nutrition education in rural regions, which is probably the reason why nutrition knowledge is higher in rural schools than in urban ones. One study found opposite results, showing that in rural schools significantly less nutrition lessons were taught than in suburban schools^(67)^. But studies regarding nutrition knowledge of children and adolescents and the number of lessons in urban and/or rural areas are scarce. It is believed that differences between urban and rural areas can vary within the respective countries, and further studies are necessary in this research field.

Furthermore, our findings illustrate a positive association between nutrition education hours per week and healthier food choices, for example, a higher intake of whole grains and a lower intake of meat and energy drinks. Current studies investigating the association of nutrition education as part of the curriculum and dietary behaviour are rare but there are numerous studies showing that nutrition interventions at school, targeting the improvement of the consumption of healthy foodstuffs such as vegetables and fruits in children and adolescents, accompanied by adjustments of the school food environment, can be successful^(65,68–74)^. Nonetheless, a review found that the lack of fidelity in peer-led interventions, lack of behaviour support for environmental interventions and too-short duration (under 5 months) were factors that contributed to the failing of some of the reviewed interventions^(75)^.

### Limitations

Some limitations of the current study need to be mentioned. First, the study design was cross-sectional. Consequently, conclusions cannot be attributed to plausible causes, but to valuable assumptions that could be used for further studies. As with any self-reported data, misreporting bias need to be taken into account. Also, it is possible that there is a tendency of respondents to answer questions in a way that will be viewed positively by others, which is called social acceptability bias. However, because of practical and budgetary reasons, this option was necessary.

Additionally, we could not provide individual assistance to the children when completing the FFQ, and therefore, we cannot exclude errors made by the children when indicating portion sizes and frequencies for each item. Thus, we refrained from drawing conclusions on the exact daily intake in grams since we think that specifications in this direction would be too vague. Therefore, for further analysis, we rather focused on consumption frequency per day and thus, more on nutrition quality than quantity. Despite their strengths of simply assessing usual dietary intake, saving time, being cost-effective and suitable for epidemiological studies, FFQ have several limitations that have to be considered. They are specific to study groups and research aims, use a closed-ended questionnaire, have low accuracy (recall bias) and require accurate evaluation^(76)^. Some studies describe an overestimation of dietary intake using an FFQ due to a large number of food items asked, offering a wider range of options, for one thing. On the other hand, increasing the frequency of food items may lead to smaller intake amounts^(77)^.

Data collection took place at the end of the school year within 4 weeks. Therefore, seasonal variations in dietary intake could not be considered. Additionally, even though the sample was representative of a typical rural and urban region in Tyrol, our sample consisted of children in a single Austrian area. Therefore, it does not permit the generalisation of the results. Lastly, the results of the Cronbach’s *α* test are low and thus considered to be insufficient. In terms of validity and reliability, the questionnaires to assess nutritional knowledge and other variables in this study need to be adjusted and tested accordingly for future investigations.

## Conclusions

Mostly, studies focus on short-term nutritional interventions, but to our knowledge not on the impact of nutrition education as part of the curriculum. Our results and those of the current literature suggest that more hours in nutrition education are somehow linked with better nutrition knowledge. However, nutrition education should not only aim to improve nutrition knowledge but also overall nutrition literacy to improve dietary and lifestyle behaviours in children and adolescents. Current data regarding the association of nutrition knowledge and dietary behaviour are very heterogenic but some clearly show a positive association between knowledge and intake of different food groups such as higher consumption of fruits and vegetables^(50,74,78)^. Children mention that education of themselves and their parents could improve awareness of the positive and negative health effects of diverse foods^(79)^. Nevertheless, there are other factors influencing food choices such as socio-demographic factors (e.g. age, gender, lower household income), peer-groups, advertising and availability. School as the primary environment of children plays a central role in education beside home and media and may compensate for social disadvantages.

Even though, with our study design, we could not consider all factors contributing to dietary behaviour, we observed the importance of regular and appropriate health education at school.

With regard to the increasing prevalence of nutrition-related diseases in children and adolescents, more hours for school-based nutrition education, nutrition-related projects and events can be seen as preventive measures to increase nutritional competences and thus, reduce disease incidence later in life. Also, schools are confronted with tremendous challenges in dealing with health and nutrition education since the role of the parents in this context has shifted due to changing rhythms of life, family structures and working conditions. Therefore, a closer look by politicians, the responsible state school councillors, municipalities and school leaders towards nutrition as an important factor in school-based health promotion is required.

Further studies are necessary to assess the quality of nutrition education to identify current problems and challenges, and in further consequence, contemporary requirements for high-quality nutrition education in Austria and other countries in order to secure the future of nutrition education in secondary schools and in other school types.
